# (*R*)-(+)-3,5-Dinitro-*N*-(1-phenylethyl)benzothioamide

**DOI:** 10.3390/m1650

**Published:** 2023-05-20

**Authors:** Matthew G. Donahue, Emily Crull

**Affiliations:** Department of Chemistry and Biochemistry, University of Southern Mississippi, Hattiesburg, MS 39406, USA

**Keywords:** benzylamine, chiral solvating agent, thioamide

## Abstract

(*R*)-(+)-3,5-dinitro-*N*-(1-phenylethyl)benzothioamide **1** is a potential chiral solvating agent (CSA) for the spectral resolution of enantiomers via ^1^H NMR spectroscopy. The single enantiomer of **1** was synthesized from commercially available (*R*)-(+)-a-methylbenzylamine **2** in two steps with 85% yield.

## 1. Introduction

**Figure 1.**
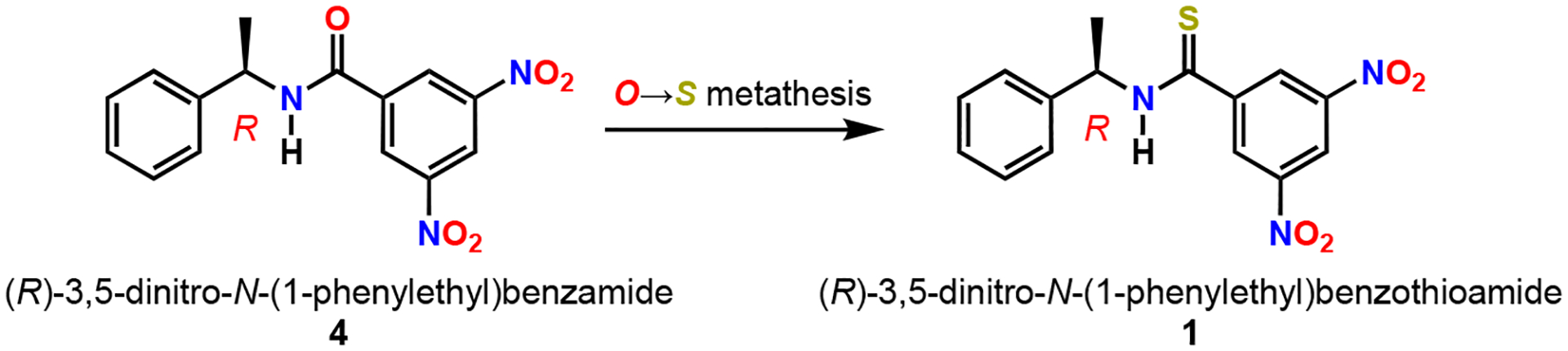
Kagan amide and thioamide chiral solvating agents.

Chiral solvating agents (CSAs) are a class of molecules utilized for the spectral resolution of enantiomers via NMR spectroscopy (Figure 1) [1]. Such a resolution is possible because the CSAs associate with analytes via non-covalent interactions (NCIs) to form diastereomeric complexes resulting in chemical shift differences ΔΔδ of the enantiomers [2]. The scaffold of the CSA must possess some stereogenic features (atom, axis, plane) embedded in its backbone along with functional groups capable of eliciting NCIs, including hydrogen bond donation/acceptance and pi acidity/basicity [3,4].

(*R*)-(−)-3,5-dinitro-*N*-(1-phenylethyl)benzamide **4**, widely known as Kagan’s amide [5], is a validated CSA for the discrimination of a wide selection of analytes with functional groups including alcohols [6], amines/amides [7], carboxylic acids, phosphine oxides [8], phospholene oxides [9], and sulfoxides. To date, the thioamide variant **1** of the Kagan amide **4** has not been disclosed in the literature. However, the desnitro compound (R)-*N*(1-phenylethyl)benzothioamide has been prepared [10], characterized in the solid state by single-crystal X-ray diffraction [11], and utilized synthetically [12]. Given the broad utility of the thiocarbonyl functional group [13] in validated organocatalysts such as thioureas [14] and thiosquaramides [15] that enable asymmetric transformations through NCIs [16,17], we hypothesized that thioamide **1** would be a competent CSA analogous to **4**. Since sulfur has a larger van der Waals radius than oxygen (S = 1.85 Å vs. O = 1.40 Å), the C=S bond is longer than the C=O bond (1.60 Å vs. 1.40 Å) [18]. Because of these physical properties, thioamides are less prone to self-aggregation than amides [19] since they are weaker hydrogen bond acceptors. Additionally, due to the increased acidity of the N–H bond of Δp*K*_a_ = −6 [20], thioamides are stronger hydrogen bond donors [21]. With these physical factors in mind, we set out to synthesize **1** for the purposes of using it as a CSA with the goal of using it as a tool for the determination of absolute configuration [22].

## 2. Results and Discussion

**Scheme 1.**
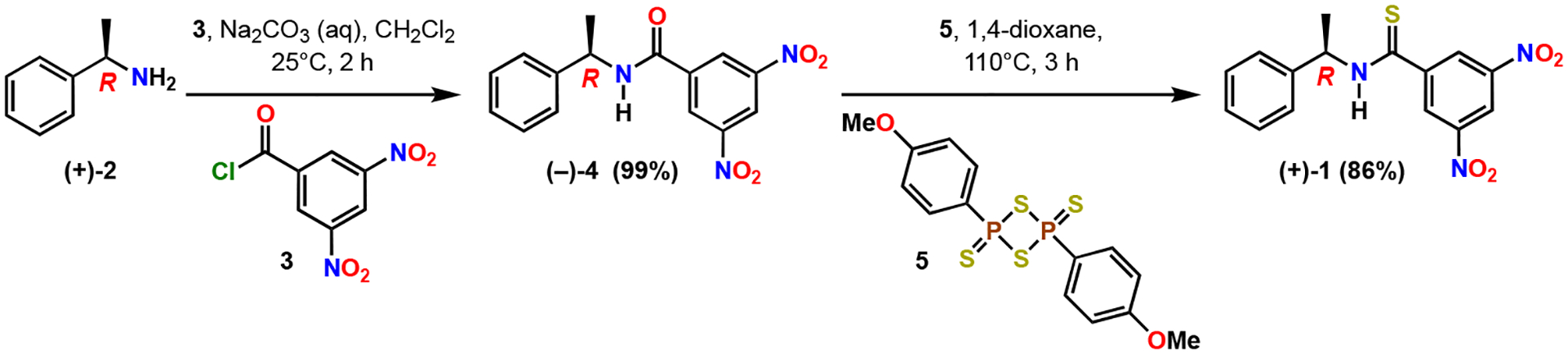
Two-step chemical synthesis of (*R*)-(+)-3,5-dinitro-*N*-(1-phenylethyl)benzothioamide (**1**).

The title compound (*R*)-(+)-3,5-dinitro-*N*-(1-phenylethyl)benzothioamide **1** was prepared in one step from (*R*)-(−)-3,5-dinitro-*N*-(1-phenylethyl)benzamide **4** (Scheme 1). The Kagan amide **4** was readily prepared in quantitative yield as an off-white solid (mp 151–153 ^◦^C) in decagram quantities through the coupling of commercially available enantiopure (*R*)-(+)-α-methylbenzylamine **2** and 3,5-dinitrobenzoyl chloride **3** under biphasic conditions with dichloromethane in aqueous sodium carbonate. The specific rotation of **4** was measured in three different solvents to be [α] −46.781 (*c* 0.873, acetone), [α] −13.540 (*c* 1.090, ethanol) and [α] −2.986 (*c* 1.007, CHCl_3_).

Amide (−)-**4** was treated with Lawesson’s thionating reagent **5** [23,24], resulting in complete conversion to the thioamide **1**. The crude ^1^H NMR showed the presence of residual aromatic impurities that mandated a relatively straightforward purification by flash column chromatography over silica gel to yield the thioamide variant **1** as bright yellow solid with mp 79–81 ^◦^C (Supplementary Material). The molecular formula of **1** was confirmed by means of high-resolution mass spectrometry to be C_15_H_13_N_3_O_4_S with m/z 354.0520 of the sodium salt.

With the confirmation that the *O*→*S* carbonyl metathesis occurred, the structure of **1** was fully elucidated using infrared and nuclear magnetic resonance spectroscopy. The thiocarbonyl stretch C=S of **1** was noticeably absent in the infrared spectrum from the typical amide C=O stretching region as observed with **4** at 1642 cm^−1^. It is known that C=S stretching lies in the 1200−1100 cm^−1^ region and is much weaker than C=O stretching [25]. While the ^13^C signal for the carbonyl carbon of **4** appeared at 161.8 ppm, the thioamide **1** shifted 29.9 ppm downfield to 191.7 ppm (Table 1). The positional assignments of carbon and hydrogen were carried out using 1D and 2D NMR techniques. The specific rotation of **1** was measured to be [α] +22.91 (c 0.965, CHCl_3_).

## 3. Materials and Methods

### 3.1. Materials

Starting materials were purchased from commercial vendors and checked for identity and purity using IR, NMR and HPLC and were used without purification unless noted. (*R*)-(+)-1-phenylethylamine (CAS# 3886–69–9) was purchased from Oakwood Chemical (Product # 037431, 99.9% ee). 3,5-Dinitrobenzoyl chloride (CAS# 99–33–2) was purchased from Oakwood Chemical (Product # 493922). Lawesson’s reagent (CAS# 19172–47–5) was purchased from Aldrich (Product # 227439).

### 3.2. Methods

Analytical thin-layer chromatography was performed using Sorbent Technologies 250 μm glass-backed UV254 silica gel plates. The plates were first visualized by means of fluorescence upon 254 nm irradiation then using an iodine chamber and subsequently with phosphomolybdic acid with heating. Flash column chromatography was performed using Sorbent Technologies 40–63 μm, pore size 60 Å silica gel in Luknova columns on a Teledyne ISCO CombiFlash Rf with solvent systems indicated. Solvent removal was effected using a Buchi R3 rotary evaporator with a V900 diaphragm pump (~10 mmHg). Further drying of samples was conducted using a Welch vacuum pump at <0 mmHg. All isolated yields refer to material that is chromatographically (TLC or HPLC) and spectroscopically (^1^H NMR) homogenous.

### 3.3. Instrumentation and Analysis

Melting points were measured on a Laboratory Devices Mel-temp with a Thermco 0–400 ^◦^C mercury thermometer (serial number 26296) using 1.5–1.8 mm O.D. tubes (Chem-Glass part number CG-1841–01) and are uncorrected. Infrared spectra were recorded on a Nicolet Nexus 470 FTIR spectrometer as neat liquids, oils, solids, or as thin films formed from the evaporation of NMR solvent over the ATR plate. Nuclear magnetic resonance spectra were measured at ambient temperature (~25 ^◦^C) on a Bruker UltraShield 400 MHz with deuterated chloroform-*d* (D,99.8% + 0.05% *v*/*v* TMS) from Cambridge Isotope Laboratories (Product # DLM-7TB). Proton nuclear magnetic resonance spectra were recorded at 400 MHz and were recorded in parts per million from internal residual protons on the scale and were reported as follows: chemical shift [multiplicity (s = singlet, d = doublet, t = triplet, q = quartet, m = multiplet), coupling constant(s) in hertz, integration, interpretation]. ^13^C NMR data were recorded at 100 MHz and were reported as follows: chemical shift with multiplicity as determined from DEPT (CH, CH_3_ up and CH_2_ down) and/or HSQC experiments. Structures were fully elucidated by assigning ^1^H peaks to their respective ^13^C peaks using the 1D and 2D NMR experiments. High-resolution mass spectra were recorded at the Old Dominion University College of Science Major Instrumentation Center (COSMIC) on a Bruker 12 Tesla APEX-Qe FTICR-MS with an Apollo II ion source. Optical rotations were nominally measured between 24 and 26 ^◦^C on a Rudolph Autopol polarimeter using a cell with a path length of 1.0 dm and a volume of 2.0 mL (part number 32–5–100–2.0). Solutions were generally prepared from approximately 0.0300 g of purified material dissolved in 3.0 mL of HPLC-grade chloroform (CAS# 67–66–3, stabilized with ethanol, Oakwood item number 101614) dispensed with a VWR Labmax solvent dispenser.

### 3.4. (R)-(+)-3,5-Dinitro-N-(1-phenylethyl)benzothioamide (1)

A 100 mL round bottom flask with a stir bar was charged with (*R*)-(−)-3,5-dinitro-*N*(1-phenylethyl)benzamide **4** (2.207 g, 7.0 mmol, 1 eq) and 1,4-dioxane (24 mL, 0.30 M) to give a pale-yellow solution. Lawesson’s reagent **5** (1.55 g, 3.85 mmol, 0.55 eq) was added resulting in a cloudy yellow mixture. The flask was equipped with a reflux condenser and a drying tube filled with Drierite and heated to 110 ^◦^C for three hours. Upon heating, the reaction became a clear dark gold color. In-process analysis via TLC (4:1 hexanes-ethyl acetate) showed many spots that were not the starting material. The starting material at R_f_ = 0.25 stained dark magenta-purple with PAA was no longer present. The solution was poured into 20 mL of cold deionized water in a 100 mL round bottom flask that was chilled in an ice water bath. The quench mixture was aged in the ice bath for 1 h. A darker gold oil separated from the mixture, but no solid formed. The solvent was concentrated in vacuo into an oil. The ^1^H NMR spectra of the crude material indicated complete conversion of the starting amide to the desired product, but the presence of multiple aromatic by-products. The material was purified by chromatography over 80 g of normal-phase silica gel using an isocratic elution of 4:1 hexanes-ethyl acetate. The product-rich fractions were pooled and concentrated to give 2.00 g (86% yield) of the title compound as a dark gold oily solid with the characterization data: MP: 79–81 ^◦^C; R_f_ = 0.35 (4:1 hexanes-ethyl acetate; uv → PAA, I_2_); IR (thin film): cm^−1^ 3342 (N-H), 1535 (NO_2_), 1340 (NO_2_); [α]^27.6^ +22.91 (*c* 0.965 g/100 mL, *D* CHCl_3_; T 27.6 ^◦^C); ^1^H NMR (CDCl_3_, 400 MHz): *δ* 8.99 (t, 1H, *J* = 2.04 Hz), 8.81 (d, 2H, *J* = 2.04 Hz), 8.12 (s, 1H), 7.29–7.43 (m, 5H), 5.83 (dq, 1H, *J* = 7.20, 7.00 Hz), 1.76 (d, 3H, *J* = 6.92 Hz); ^13^C{^1^H} NMR (CDCl_3_, 100 MHz): _*δ*_ 191.7 (s), 148.1 (s), 144.5 (s), 140.4 (s), 129.0 (d), 128.3 (d), 126.8 (d), 126.7 (d), 119.9 (d), 56.2 (d), 19.9 (q); HRMS (ESI): Exact mass calcd for C_15_H_13_N_3_O_4_S [M+Na]^+^ *m*/*z* 354.0518. Found *m*/*z* 354.0520.

## 4. Conclusions

The treatment of the Kagan amide (−)-**4** with Lawesson’s reagent **5** in 1,4-dioxane effected the smooth transformation to the thioamide (+)-**1** with 85% yield on a multi-gram scale. Given the commercial availability of all of the reagents at relatively inexpensive cost, this method is a viable route to obtain the sulfur derivative of the common chiral solvating agent.

## Supplementary Material

SI Data

## Figures and Tables

**Table 1.** Structural assignments using ^1^H (400 MHz) and ^13^C (100 MHz) NMR data of (−)-**4** and (+)-**1** in CDCl3

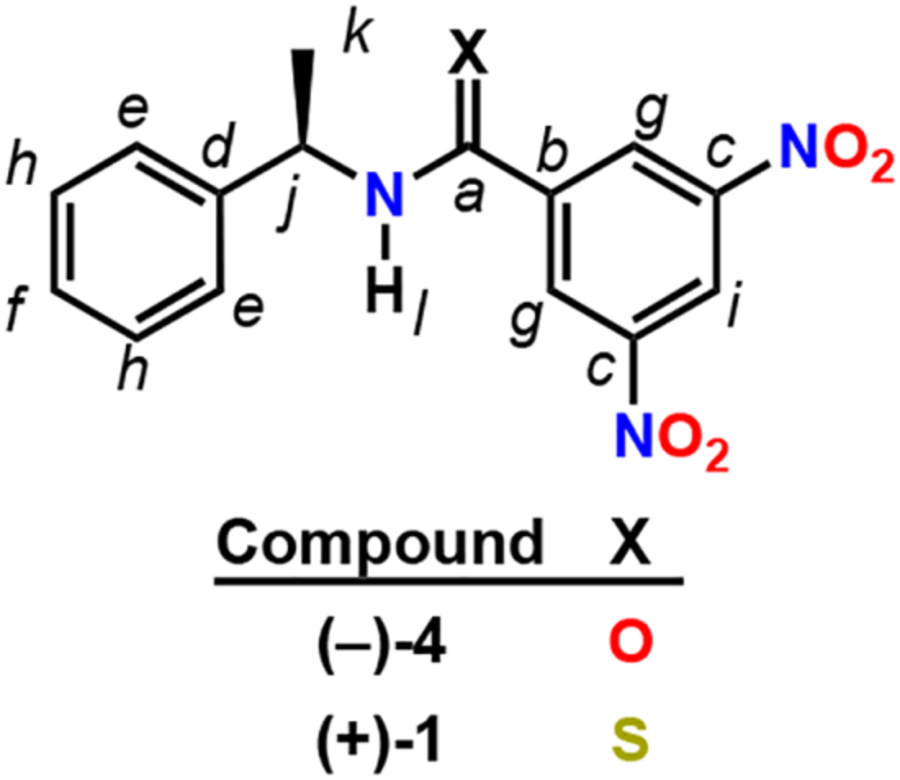				
	^13^C NMR Data^1^		1H NMR IData^2^	
Position^3^	4 δ_C_	1 δ_C_	4 δ_H_	1 δ_H_
a	161.8 (s)	191.7 (s)	-	-
b	148.6 (s)	144.5 (s)	-	-
c	141.8 (s)	148.1 (s)	-	-
d	137.9 (s)	140.4 (s)	-	-
e	129.0 (d)	129.0 (d)	7.41–7.35 (m, 2H)	7.43–7.29 (m, 2H)
f	128.0 (d)	128.3 (d)	7.33–7.28 (m, 1h)	7.43–7.29 (m, 1H)
g	127.1 (d)	126.7 (d)	8.93 (d, J = 2.04 Hz, 2H)	8.81 (d, *J* = 2.04 Hz, 2H)
h	126.3 (d)	126.8 (d)	7.41–7.35 (m, 2H)	7.43–7.29 (m, 2H)
i	121.1 (d)	119.9 (d)	9.14 (t, J = 2.08 Hz, 1H)	8.99 (t, J = 2.04 Hz, 1H)
j	50.3 (d)	56.2 (d)	5.34 (dq, J = 7.16, 7.12 Hz, 1H)	5.83 (dq, J = 7.20, 7.00 Hz, 1H)
k	21.4 (q)	19.9 (q)	1.67 (d, J = 6.92 Hz, 3H)	1.76 (d, J = 6.92 Hz, 3H)
l	-	-	6.64 (s, 1H)	8.12 (s, 1H)

1 13C NMR signal multiplicity determined by DEPT90, DEPT135 and ^1^H-^13^C HSQC.

2 Integration and multiplicity determined by 1H NMR and *J*-value coupling analysis.

3 Positions assigned using ^1^H-^1^H COSY, ^1^H-^13^C HSQC, and ^1^H-^13^C HMBC analysis.

## Data Availability

Data are contained within the article or Supplementary Material.
