# Supplementary material for: (R)-(+)-3,5-Dinitro-N-(1-phenylethyl)benzothioamide
Source: Molbank. Author manuscript; Available in PMC 2024 Jan 25. (PMC10810360; doi:10.3390/m1650)
Supplement: SI Data [file NIHMS1960482-supplement-SI_Data.pdf]

thermo
scientific

EBC-6-067-1 8/7/2020 12:29:54 PM

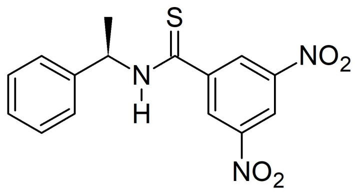

3,5-dinitro-*N*-[(1*R*)-1-phenylethyl]
benzene-1-carbothioamide
InChI Key: JQRKUKGQZCQJHX-SNVBAGLBSA-N

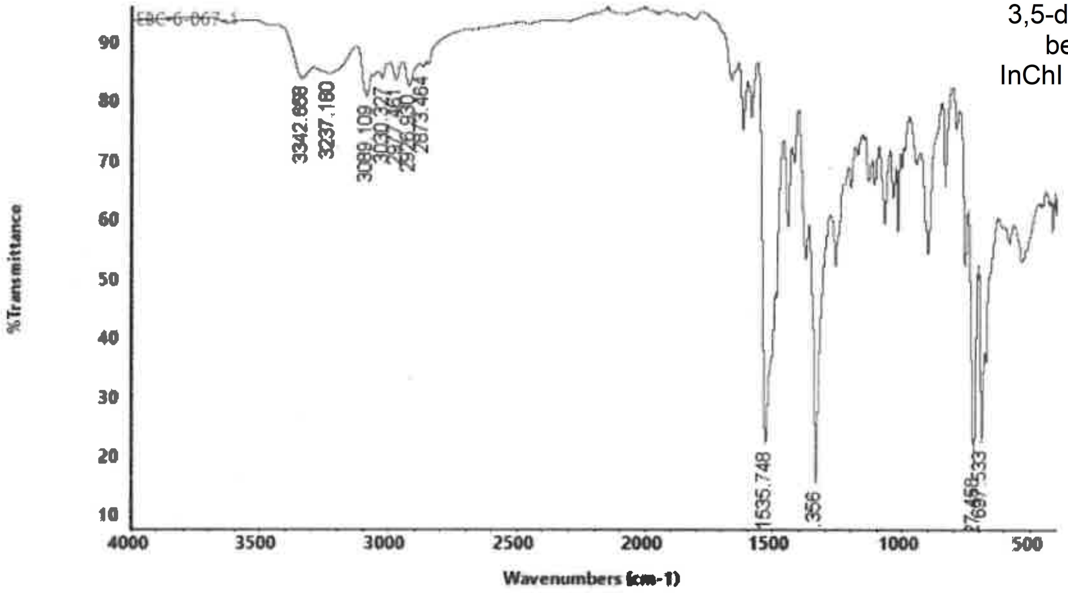

Title: EBC-6-067-1
Number of sample scans: 16
Number of background scans: 16
Instrument Serial: BDM1910286
Smart Accy: V70520
Model: Summit
Source: IR
Detector: DTGS KBr
Smart Accessory Title: Everest ATR
Smart Accessory ID: V70520
Crystal type: : None
Beamsplitter: KBr
Sample spacing: 1.0
Digitizer bits: 24
Optical velocity: 0.4747
Aperture: 100.0
Sample gain: 1.0
High pass filter: 1.0
Low pass filter: 11000.0
Comments: None

Regions:

Region 1: 3477.39-2805.65
Threshold: 87.29
Sensitivity: 50.00

Region 2: 1675.05-582.28
Threshold: 40.44
Sensitivity: 50.00

Position	Intensity
2873.46	85.53
2926.93	82.43
2977.46	83.43
3030.33	83.72
3089.11	80.54
3237.18	84.62
3342.66	83.68
697.53	22.27
727.46	17.73
1340.36	15.42
1535.75	22.00

*1 **1H NMR** (CDCl3, 400 MHz)*

NAME	EBC-6-067-1
EXPNO	10
PROCNO	1
Date_	20200714
Time	22.14 h
INSTRUM	spect
PROBHD	Z108618_0161 (
PULPROG	zg30
TD	65536
SOLVENT	CDCl3
NS	16
DS	2
SWH	8012.820 Hz
FIDRES	0.244532 Hz
AQ	4.0894966 sec
RG	90.5
DW	62.400 usec
DE	6.50 usec
TE	297.9 K
D1	1.00000000 sec
TD0	1
SFO1	400.1724710 MHz
NUC1	1H
P1	9.88 usec
SI	65536
SF	400.1700120 MHz
WDW	EM
SSB	0
LB	0.30 Hz
GB	0
PC	1.00

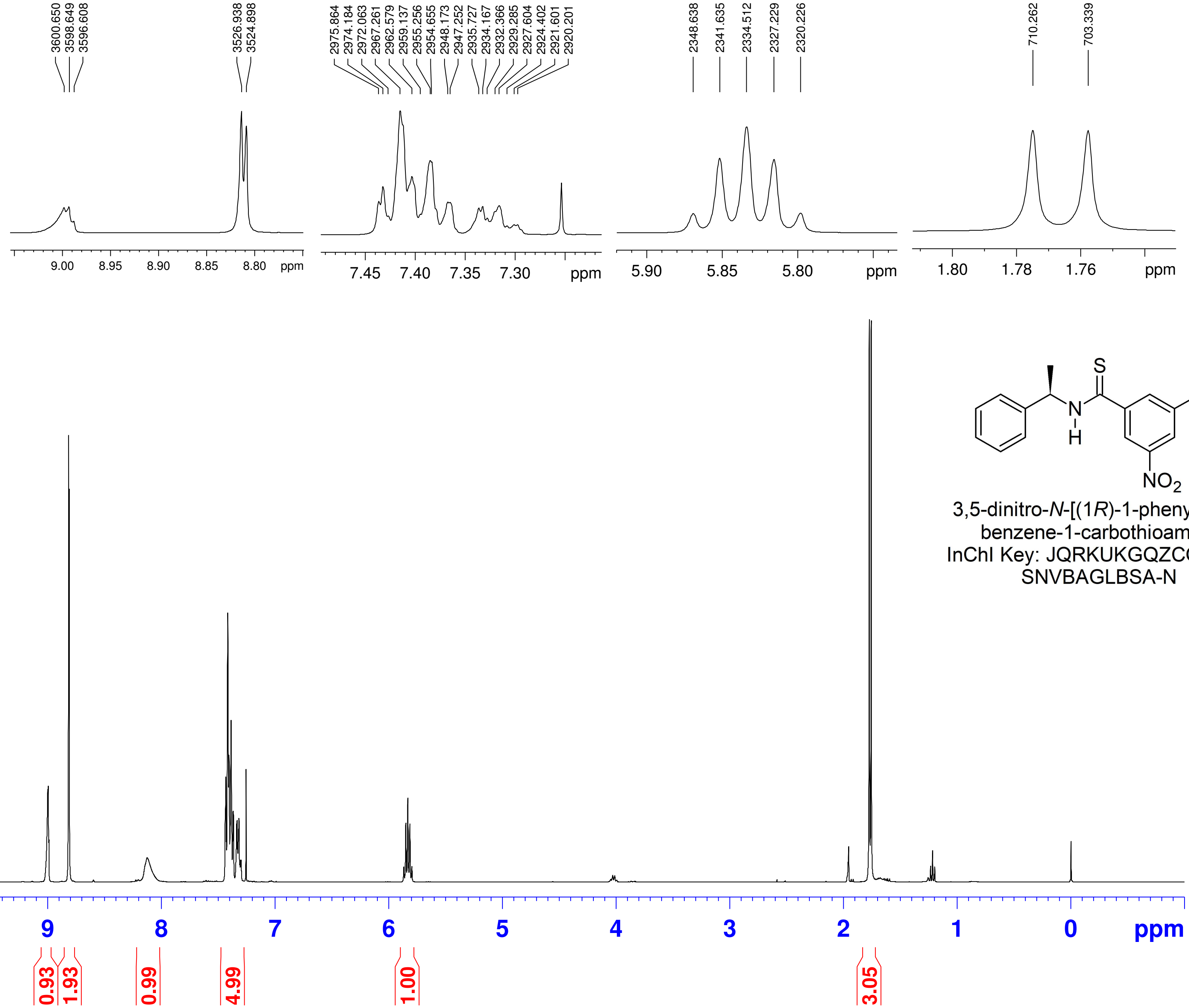

3,5-dinitro-*N*-[(1*R*)-1-phenylethyl] benzene-1-carbothioamide
InChI Key: JQRKUKGQZCQJHX-SNVBAGLBSA-N

11H-1H COSY(CDCl3, 400 MHz)

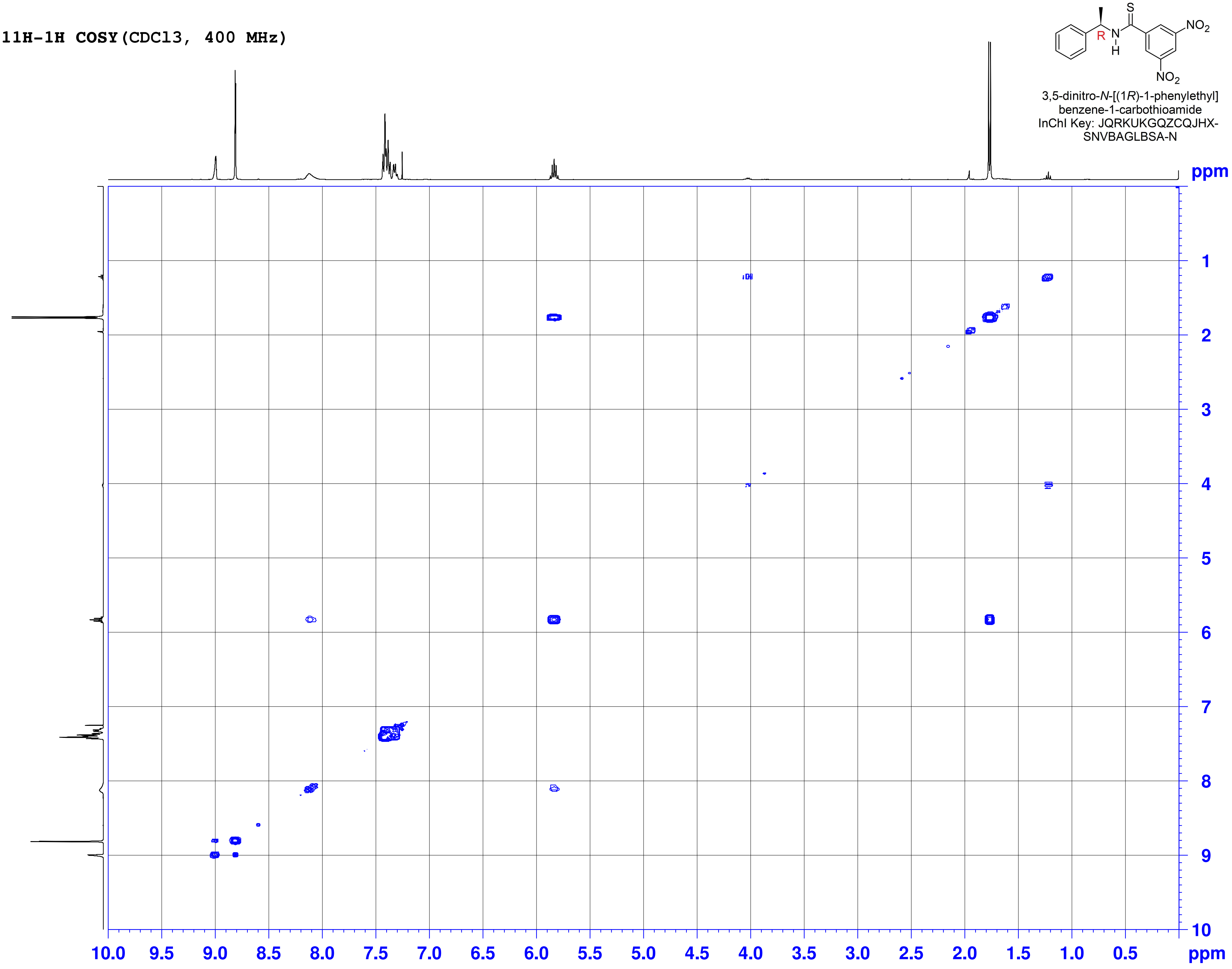

1 13C NMR (CDCl3, 400 MHz)

NAME	EBC-6-067-1
EXPNO	12
PROCNO	1
Date_	20200714
Time	23.31 h
INSTRUM	spect
PROBHD	Z108618_0161 (
PULPROG	deptsp135
TD	65536
SOLVENT	CDCl3
NS	256
DS	8
SWH	24038.461 Hz
FIDRES	0.733596 Hz
AQ	1.3631988 sec
RG	203
DW	20.800 usec
DE	6.50 usec
TE	298.5 K
CNST2	145.0000000
D1	2.00000000 sec
D2	0.00344828 sec
D12	0.00002000 sec
TD0	1
SFO1	100.6328888 MHz
NUC1	13C
P1	8.40 usec
P13	2000.00 usec
SI	32768
SF	100.6228265 MHz
WDW	EM
SSB	0
LB	1.00 Hz
GB	0
PC	1.40

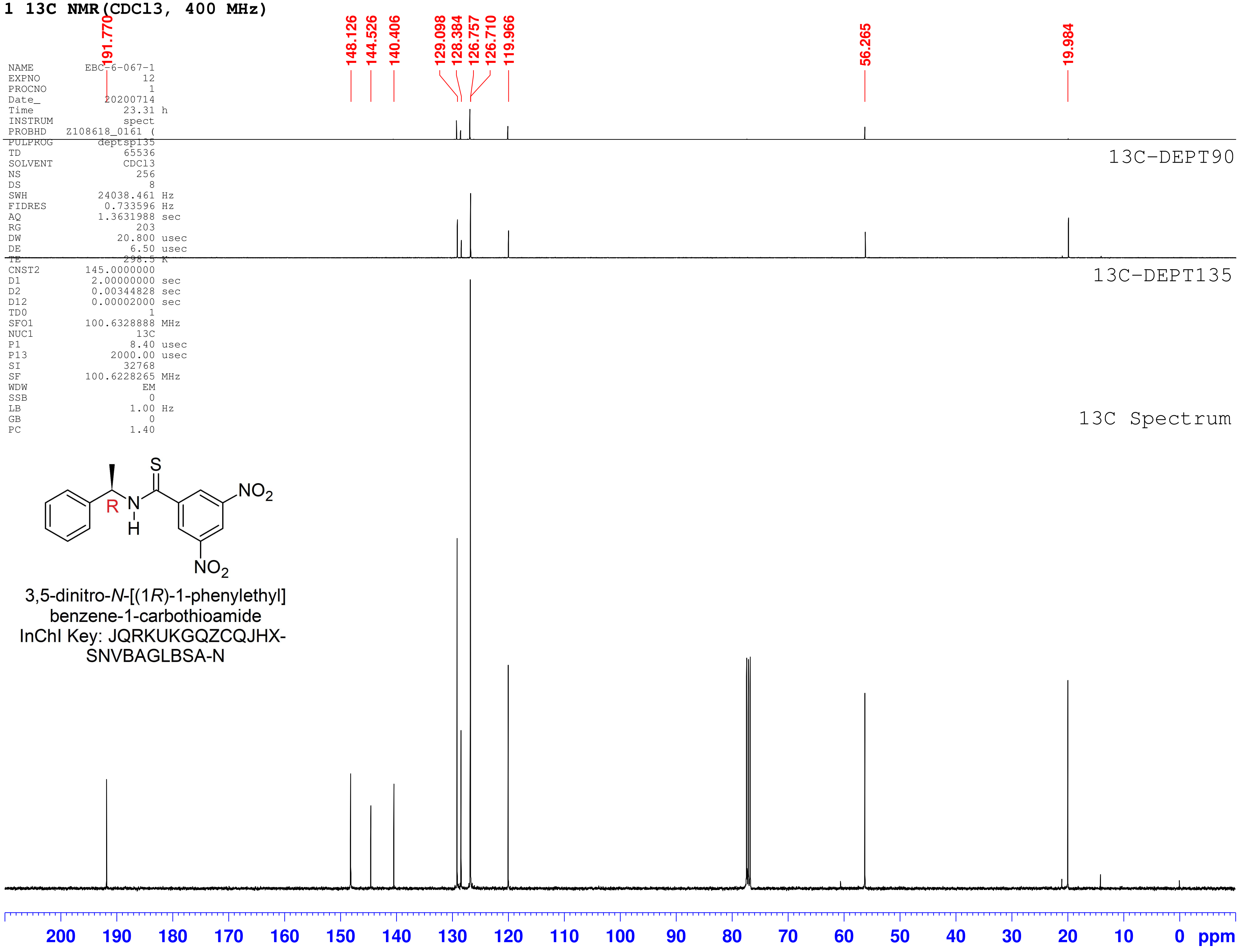

3,5-dinitro-*N*-[(1*R*)-1-phenylethyl]
benzene-1-carbothioamide
InChI Key: JQRKUKGQZCQJHX-SNVBAGLBSA-N

1 1H-13C HSQC(CDCl3, 400 MHz)

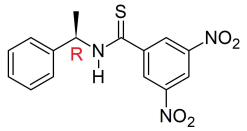

3,5-dinitro-*N*-[(1*R*)-1-phenylethyl]
benzene-1-carbothioamide
InChI Key: JQRKUKGQZCQJHX-
SNVBAGLBSA-N

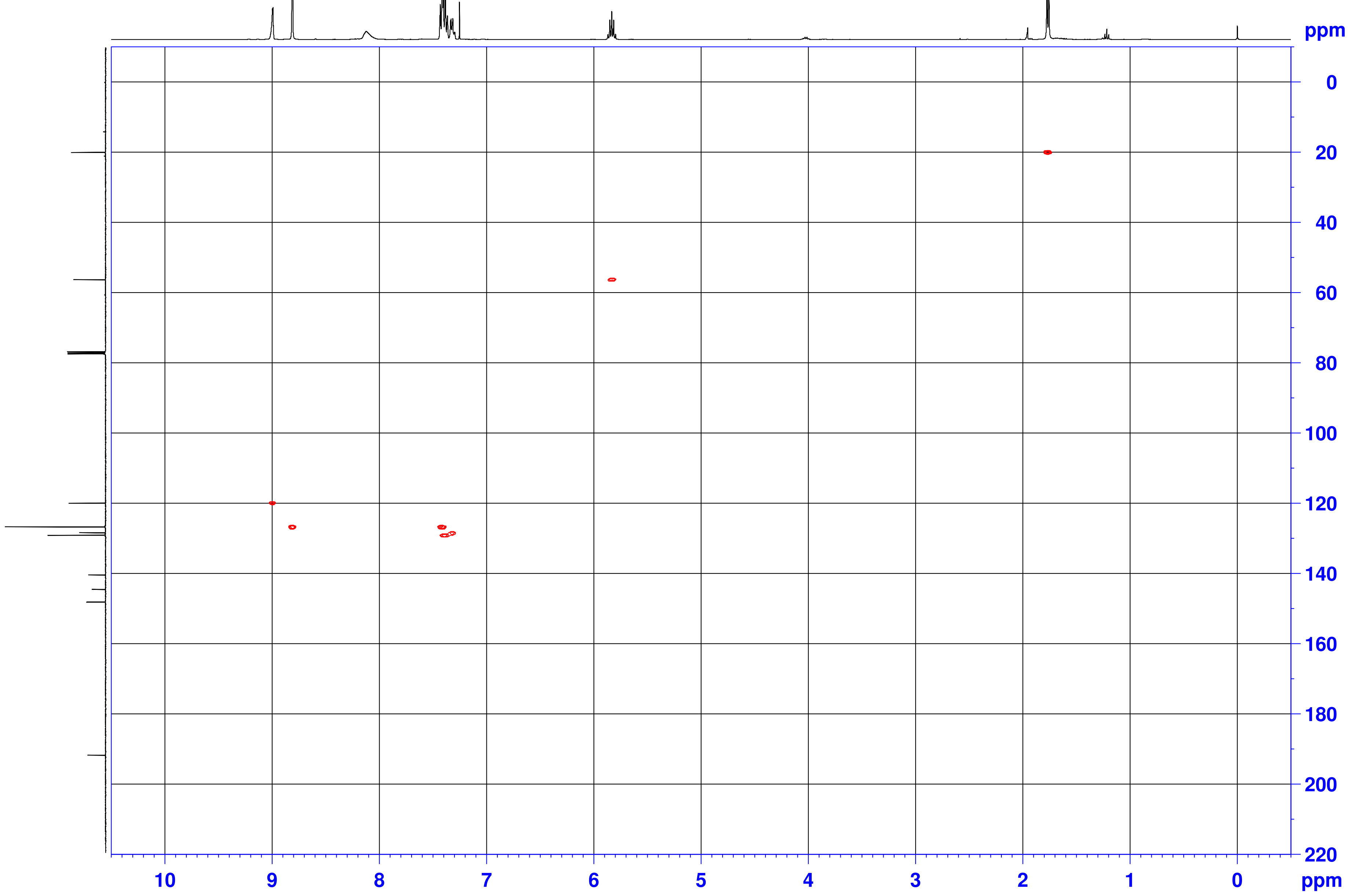

1 1H-13C HMBC(CDCl3, 400 MHz)

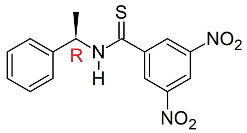

3,5-dinitro-*N*-[(1*R*)-1-phenylethyl]
benzene-1-carbothioamide
InChI Key: JQRKUKGQZCQJHX-
SNVBAGLBSA-N

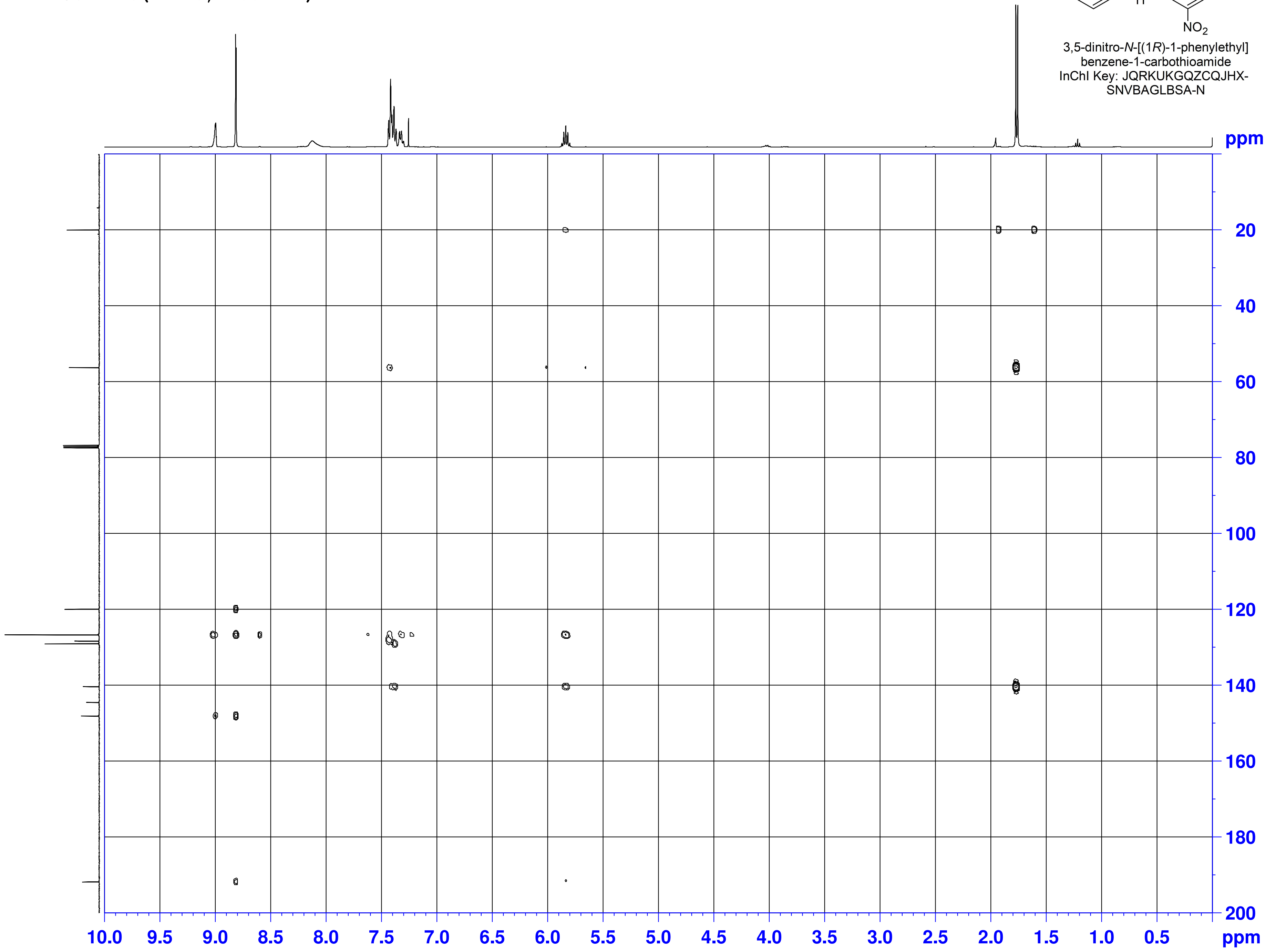

Mass Spectrum List Report

Analysis Info

Analysis Name	Z:\FTICR-MS\MS-1\Data\2020\apexdata082820\EBC-6-067-1_pos_000001.d	Acquisition Date	8/28/2020 4:45:57 PM
Method		Operator	FTMS_USER
Sample Name	EBC-6-067-1	Instrument	apex-Qe
Comment	EBC-6-067-1 in MeOH C15H13N3O4S Na+		

Sample Name EBC-6-067-1 in MeOH

Exact Mass C15H13N3O4S Na+ = 354.051898 m/z

Mass Observed = 354.052030 m/z

Difference < 1.0 ppm

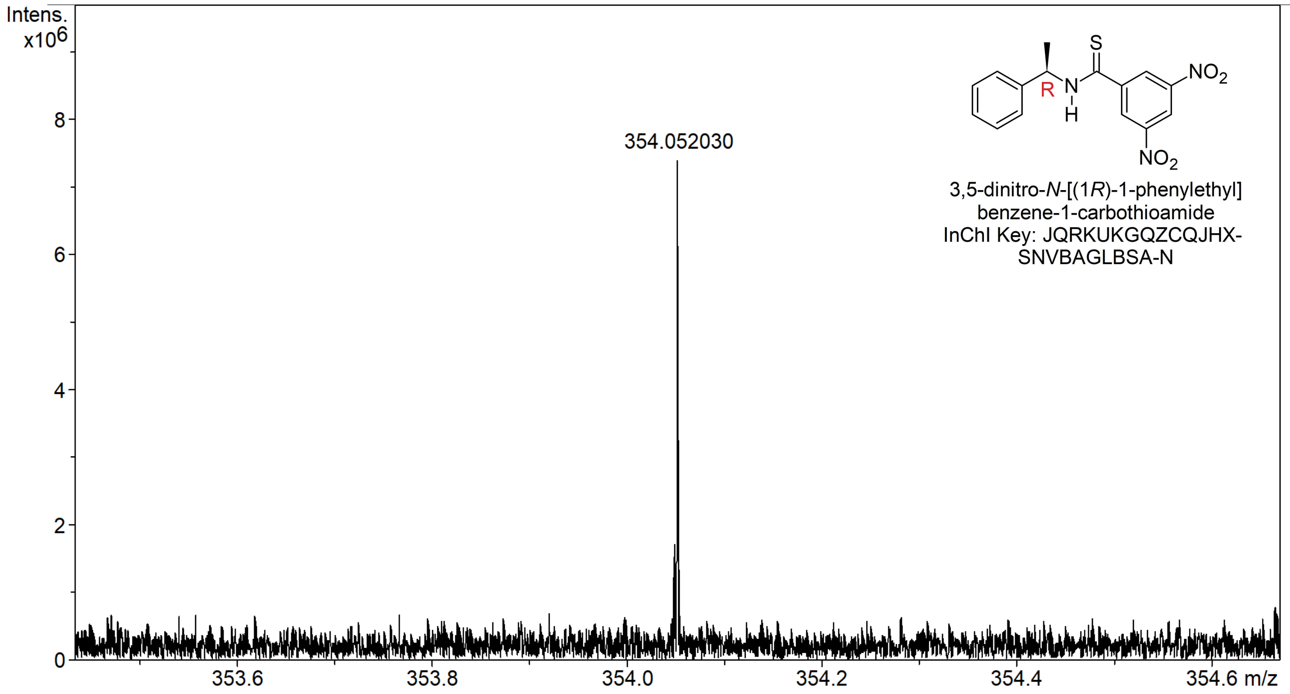

#	m/z	I
1	141.347114	1522262
2	144.081461	1496010
3	149.802439	1553179
4	153.427171	2880642
5	153.459238	1511076
6	154.450270	3418986
7	174.369974	13477814
8	175.927352	4470211
9	196.467440	1774035
10	196.779641	1755750
11	197.303609	1714064
12	197.429094	1729869
13	197.429994	1974800
14	197.462174	1803937
15	197.472420	1737659
16	198.017662	1757629
17	198.289467	1900684
18	198.308615	1752380
19	198.362267	2222468
20	198.378761	1783726

Mass Spectrum List Report

#	m/z	I
21	198.684229	1750163
22	198.753977	1884707
23	198.783278	2314541
24	198.788836	1809851
25	198.799065	1850069
26	198.813180	1734201
27	198.863345	1796279
28	198.889956	1980411
29	198.901805	1794009
30	198.910825	1902896
31	198.925340	1819604
32	198.957561	1895142
33	199.448898	1844161
34	199.472875	1771517
35	199.478010	2148362
36	199.493778	2006194
37	199.496363	1953273
38	199.532316	2049300
39	199.544306	1762034
40	199.728335	1759875
41	199.891507	1847456
42	205.461094	1823119
43	205.702015	1997278
44	206.092505	1856986
45	206.643907	1885786
46	206.902345	1823181
47	206.927910	1817483
48	206.988507	1949342
49	207.375095	1943171
50	207.431120	1984714
51	207.438432	1928412
52	207.490337	2074905
53	207.491498	2121948
54	207.528165	1829684
55	207.539521	2210639
56	207.543512	1847129
57	207.564625	1885707
58	207.570376	1848473
59	208.187513	1857759
60	208.192862	1899628
61	212.481491	2343574
62	212.482972	2721049
63	212.485955	1982368
64	212.487277	3580323
65	214.257808	2088754
66	216.557100	2147404
67	216.612020	1951930
68	216.700619	1903851
69	216.839332	2046784
70	216.858642	2014886
71	216.859800	1947944
72	217.437422	1903770
73	217.466496	1968595
74	217.650946	1920057
75	217.792262	2036939
76	262.090769	2210325
77	263.004357	2026288
78	263.076856	1902303
79	263.419985	2148957
80	264.218419	1932743
81	265.162570	1986149
82	265.219428	2062368
83	265.254678	1987317
84	265.304144	2207545

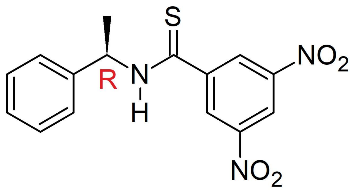

3,5-dinitro-*N*-[(1*R*)-1-phenylethyl]
benzene-1-carbothioamide
InChI Key: JQRKUKGQZCQJHX-SNVBAGLBSA-N

Mass Spectrum List Report

#	m/z	I
85	265.343919	2004617
86	265.531329	2037796
87	265.561466	1906559
88	265.570000	2252660
89	265.572528	2174577
90	275.998638	5727208
91	284.331227	13858511
92	285.334671	2803567
93	288.636284	1774976
94	288.667864	1840962
95	288.688558	1807385
96	288.702298	1796605
97	288.704368	2238202
98	288.732708	2080194
99	288.792246	1843788
100	290.312254	1767290
101	290.681372	1785492
102	290.896472	1849314
103	292.228509	1801935
104	292.883047	1791376
105	292.904314	1902309
106	293.268390	1766646
107	294.116888	1730071
108	295.139512	1705022
109	295.343707	2070290
110	295.383807	1857981
111	295.410737	1726341
112	295.445005	1830460
113	295.479939	1805811
114	295.831442	1731598
115	295.952743	1718158
116	296.164533	1811279
117	296.279669	1919323
118	296.398117	2013536
119	296.435638	1964048
120	296.440027	1704967
121	296.468432	1739979
122	296.659409	1661494
123	296.696446	1791975
124	296.817042	1902823
125	297.216544	1761684
126	297.313548	1656773
127	297.484629	1743960
128	297.602765	1783131
129	297.683382	1683375
130	297.819259	1909644
131	297.901773	1717083
132	297.932090	1822618
133	297.980310	1663411
134	298.126136	1703674
135	298.180465	1995653
136	298.351166	1690135
137	298.357099	1787914
138	298.380970	1720534
139	298.416654	1689738
140	298.655871	1663623
141	298.681946	2002127
142	298.683842	1944395
143	298.709713	1755027
144	298.740191	1703121
145	298.746856	1689667
146	298.777807	1813120
147	304.299996	3242296
148	308.210046	1553401

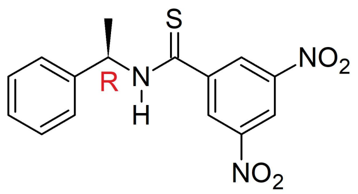

3,5-dinitro-*N*-[(1*R*)-1-phenylethyl]
benzene-1-carbothioamide
InChI Key: JQRKUKGQZCQJHX-
SNVBAGLBSA-N

Mass Spectrum List Report

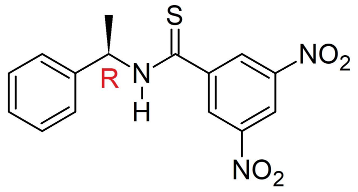

3,5-dinitro-*N*-[(1*R*)-1-phenylethyl]
benzene-1-carbothioamide
InChI Key: JQRKUKGQZCQJHX-
SNVBAGLBSA-N

#	m/z	I
149	308.420610	1557277
150	314.030708	1497787
151	314.840435	1522429
152	314.859347	1549009
153	314.915376	1776209
154	314.927032	1476150
155	317.563709	1463448
156	317.666744	1541548
157	324.077898	2509650
158	325.878807	1755983
159	330.481559	1372868
160	332.070068	1350768
161	332.331221	2594477
162	337.439951	1480330
163	337.852251	1286548
164	338.074868	4096439
165	339.704222	1314599
166	340.051753	6399816
167	340.398568	1585517
168	341.055025	1288202
169	353.266183	1228946
170	354.048820	1780974
171	354.052030	7500520
172	355.055289	1614786
173	360.323793	1925069
174	365.105660	1379742
175	368.425148	1878055
176	370.026018	3782458
177	370.046979	5544220
178	371.315905	1044546
179	372.243729	995168
180	385.292625	1371340
181	386.399365	1177373
182	391.284457	1005600
183	393.209706	1308585
184	393.297716	9434745
185	393.308830	1171571
186	394.301047	2279015
187	395.658278	926302
188	398.241543	1006313
189	401.266512	1257130
190	402.357969	1784277
191	403.999925	1901287
192	406.158537	1733692
193	408.308565	2348069
194	409.183603	3692611
195	409.271634	5199943
196	410.275074	1169286
197	413.266368	6009309
198	414.215435	1363318
199	414.269741	1766527
200	414.430603	2194587
201	415.254044	953140
202	418.015721	1373587
203	421.328907	1186323
204	424.019227	982354
205	424.282526	1372839
206	426.357821	1198683
207	428.373592	2436628
208	429.240189	3064645
209	429.318787	2512225
210	430.389296	1960669
211	433.380563	3872002
212	434.383930	2016867
